# Brachioradial Pruritus: Clinical, Electromyographic, and Cervical MRI Features in Nine Patients

**DOI:** 10.7759/cureus.21811

**Published:** 2022-02-01

**Authors:** Lisa B Shields, Vasudeva G Iyer, Yi Ping Zhang, Christopher B Shields

**Affiliations:** 1 Neurological Surgery, Norton Neuroscience Institute, Norton Healthcare, Louisville, USA; 2 Neurology, Neurodiagnostic Center of Louisville, Louisville, USA

**Keywords:** cervical radiculopathy, cervical mri, electrodiagnostic studies, brachioradial pruritus, neurology

## Abstract

Background

Brachioradial pruritus (BRP) is a neuropathic dysesthesia manifesting as pruritus over the dorsolateral forearm. While the etiology is unknown, intensive sun exposure and cervical spine disease have been proposed. This study describes the clinical, electrodiagnostic (EDX), and cervical MRI findings in nine patients diagnosed with BRP.

Materials and methods

All patients underwent EDX and cervical MRIs. Numerous metrics were documented including presenting symptoms, neurological examination, EDX findings, and cervical MRI features.

Results

All nine patients experienced pruritus of the arms/forearms, typical of BRP, which was unilateral in eight (89%) cases. Decreased pinprick sensation was noted in the arms/forearms (five [56%] patients) or of the thumbs, index, and/or middle fingers (four [44%] patients). Four (44%) patients had either decreased or absent biceps and brachioradialis deep tendon reflexes (DTRs), while one (11%) patient had decreased triceps and brachioradialis DTRs. The EDX revealed abnormalities in eight (89%) patients. Increased polyphasic units, decreased motor units, and/or denervation changes were recorded by needle electromyography (EMG) in eight (89%) patients: the biceps in seven (88%) and both the brachioradialis and triceps in four (50%) patients. The EMG abnormalities indicated chronic radiculopathy involving C6 in six patients and C5 and C6 in one patient. All nine patients had cervical spine disease, encompassing disc protrusions, spondylosis, spinal stenosis, and/or foraminal stenosis.

Conclusions

BRP in this series of patients was accompanied by chronic cervical radiculopathy involving predominantly C6 and C5. EDX and cervical spine MR imaging should be considered essential investigations in the evaluation of patients with BRP.

## Introduction

Brachioradial pruritus (BRP) is a neuropathic pruritus marked by itching, burning, stinging, and tingling over the dorsolateral aspect of the arms and forearms [1-8]. The pruritus is often intense, paroxysmal, and unrelieved by scratching; patients often resort to using icepacks to get relief. While some patients may not have cutaneous abnormalities, others may experience skin excoriations or lichenification caused by the scratching [1, 3, 8]. Women are affected in 70% of cases, primarily in the 5th and 6th decade [4, 9]. The frequency of BRP in the general population is unknown and considered very rare. Most physicians are unfamiliar with this condition, hence, the true prevalence is not known. Initially described as “solar pruritus” by Waisman in 1968, the pathology was originally considered to be related to intense sun exposure and was characterized by summer recurrences and exacerbations [10]. In 1983 Heyl described cervical vertebral osteoarthritis in patients with BRP, suggesting the association between BRP and cervical nerve compression due to cervical spine lesions [11]. BRP most commonly involves C5 and C6 radiculopathy and is bilateral in 75% of cases [4, 7, 9].

The diagnosis of BRP may prove challenging as patients often undergo invasive skin biopsies and are evaluated by various specialists, most frequently by dermatologists as well as neurologists and pain medicine physicians [9]. The “ice pack sign” is pathognomonic for diagnosis, as the pruritus is alleviated when an ice pack is placed with a subsequent return of symptoms when it is removed [8, 9]. Electrodiagnostic studies (EDX) have been rarely used in the diagnostic evaluation of BRP, however, they may reveal electromyographic changes suggestive of a cervical radiculopathy [12-14]. Treatment of BRP with topical corticosteroids, oral antihistamines, and anti-inflammatories are often ineffective, while capsaicin cream, gabapentin, pregabalin, amitriptyline, ketamine, lamotrigine, and intradermal injections of botulinum toxin (BoNT) type A have proven more beneficial [1-5, 9, 13, 15]. Pereira et al. reported normalization of reduced keratinocytic expression of TRPV1 in areas of involved skin after three weeks of treatment with capsaicin 8% [16].

We report nine patients who were diagnosed with BRP, all of whom underwent EDX and a cervical MRI. The presenting symptoms, physical examination, cervical MRI, and EDX findings in these nine patients are featured. The possible association between BRP and cervical spine pathology and treatment is discussed. The pathophysiology of BRP and the importance of EDX in the diagnostic evaluation of BRP are also discussed.

## Materials and methods

Under an Institutional Review Board (IRB)-approved protocol, we performed a 10-year (2012-2021) retrospective analysis of patients referred to our Neurodiagnostic Center for EDX studies to evaluate the presence of upper limb neuropathy such as carpal tunnel syndrome or cervical radiculopathy. Over this 10-year period, approximately 15,000-20,000 patients were referred to our facility for EDX studies. The EDX physician diagnosed the nine patients with BRP based on the pre-test clinical evaluation. Except in one patient, the referring physician did not consider a diagnosis of BRP. The patients underwent our protocol of an initial neurological examination followed by nerve conduction and electromyography (EMG) studies. BRP was not diagnosed based on the EDX studies. The EDX studies and cervical MRIs were used to determine whether the patients with BRP had underlying cervical radiculopathy (C5-C6 nerve roots which supply the area of pruritus).

The clinical history evaluated the onset and progression of symptoms, with identification of paroxysms of severe pruritus involving the dorsolateral forearm and or upper arm. Details regarding the nature and topography of sensory symptoms including pruritus were obtained, including triggering/worsening and relieving factors. The presence and distribution of cervical radicular symptoms were documented. The neurological examination was aimed at mapping out the area of sensory disturbance (pain, light touch, 2-point discrimination, temperature, position, and vibration senses) along with the evaluation of muscle strength, tone, and reflexes. The presence of skin lesions secondary to pruritus was also assessed.

The EDX studies were performed in our American Association of Neuromuscular & Electrodiagnostic Medicine (AANEM)-accredited facility using standard protocol of our laboratory [17]. Motor conduction studies were performed on median, ulnar, and radial nerves. Sensory conduction studies included the median, ulnar, lateral antebrachial cutaneous, and superficial radial nerves. The needle EMG study was performed on the brachioradialis, biceps, deltoid, triceps, pronator teres, and cervical paraspinal muscles looking for denervation/reinnervation changes at the C5, C6, and C7 myotomes. The cervical spine MRIs included T1- and T2-weighted images.

Informed consent was obtained from all patients. The University of Louisville IRB determined that our study was exempt according to 45 CFR 46.101(b) under Category 4. The IRB number is 21.0796.

## Results

Clinical findings and neurological examination of patients with brachioradial pruritus

Nine patients were diagnosed with BRP based on presenting symptoms (paroxysms of pruritus in the dorsolateral forearm and or upper arm), neurological examination, and EDX findings (Table 1).

**Table 1:** Patients with brachioradial pruritus evaluated at our electrodiagnostic center DOC: disc-osteophyte complex

Patient #	Age (years)/ Gender	Presenting Symptoms	Neurological Examination	Cervical MRI	Electrodiagnostic Studies
1	59/F	Pruritus right radial forearm/radial 2 fingers	Decreased pinprick sensation right radial 3 digits; absent right brachioradialis and biceps reflexes	Anterior cervical fusion C5-6; bilateral foraminal narrowing C4-5	Increased neuropathic polyphasic units right brachioradialis, biceps, triceps
2	50/F	Pruritus bilateral shoulders to elbows	Decreased pinprick sensation bilateral radial upper arms, lateral forearms; diminished left biceps reflex and bilateral brachioradialis reflexes	Bilateral neuroforaminal stenosis C3-4, C4-5, C5-6	Increased neuropathic polyphasic units left deltoid, biceps, brachioradialis
3	62/F	Pruritus left distal upper arm/lateral forearm	Wasting of left deltoid, infraspinatus, biceps, pronator teres; decreased pinprick sensation radial left forearm; absent left biceps and brachioradialis reflexes	Disc protrusion, spondylosis, spinal stenosis, and foraminal stenosis C3-7	Decreased motor units and increased neuropathic polyphasic units left infraspinatus, deltoid, supraspinatus, biceps, brachioradialis, pronator teres
4	63/F	Pruritus/tingling radial left forearm, thumb, index finger	Decreased pinprick sensation left thumb	Multilevel spondylosis, greatest at C5-6 with DOC causing canal stenosis, cord flattening, foraminal narrowing	Increased neuropathic polyphasic units left deltoid, biceps, brachioradialis
5	50/F	Pruritus left upper arm	Decreased pinprick sensation radial 3 digits bilaterally	DOC C4-5, C5-6, C6-7	No denervation or reinnervation changes C5-T1
6	54/F	Pruritus left upper arm	Decreased pinprick sensation radial left forearm	DOC, spinal stenosis, foraminal stenosis C5-6	Increased neuropathic polyphasic units left biceps, triceps
7	47/M	Pruritus radial right forearm	Decreased pinprick sensation radial right forearm/wrist; decreased brachioradialis and triceps reflexes bilaterally	Neuroforaminal narrowing C5-6, C6-7	Fibrillations and neuropathic polyphasic units Denervation of in bilateral triceps and C6-7 paraspinals
8	62/M	Pruritus and tingling lateral left forearm	Decreased pinprick sensation lateral distal left forearm; absent left biceps and brachioradialis reflexes	DOC, neuroforaminal narrowing, cord flattening C3-4, C4-5	Increased neuropathic polyphasic units left deltoid, biceps
9	71/F	Pruritus radial right forearm	Decreased pinprick sensation thumbs bilaterally	Disc protrusions, degenerative spondylosis resulting in spinal stenosis C4-5, C5-6; foraminal stenosis C3-4, C4-5, C5-6, most severe at C5-6 impinging on left C6 nerve root	Increased neuropathic polyphasic units in deltoid, biceps, triceps bilaterally

BRP was suspected in only one of these patients prior to the referral for EDX. The mean age was 57.5 years (range: 47-71 years), and seven (78%) patients were female. All nine patients complained of severe pruritus of the upper extremities which was unilateral in eight (89%) cases. None of the patients had radicular pain on presentation; two patients had a radicular tingling sensation along with the intense pruritus. The patients did not report any predisposing or inciting factors such as prolonged sun exposure prior to the BRP diagnosis, however, they often stated that the symptoms were worse in the summer than in winter.

Decreased pinprick sensation was noted in the arms/forearms (five [56%] patients) or in the thumbs, index, and/or middle fingers (four [44%] patients) (Table 1). Four (44%) patients had either decreased or absent biceps and brachioradialis deep tendon reflexes (DTRs), while one (11%) patient had decreased triceps and brachioradialis DTRs.

Electrodiagnostic studies in patients with brachioradial pruritus

Increased polyphasic units (large amplitude, wide duration), decreased motor units, and/or denervation changes were recorded by needle EMG in eight (89%) patients: the biceps in seven (88%) patients and both the brachioradialis and triceps in four (50%) patients. The EMG abnormalities were consistent with chronic radiculopathy involving C6 in six patients and at C5 and C6 in one patient.

Cervical MRI findings in patients with brachioradial pruritus

All nine patients had evidence of cervical spine disease, encompassing disc protrusions, spondylosis, spinal stenosis, and/or foraminal stenosis (Table 1). Only one (11%) patient had single-level cervical pathology, while the remainder had multi-level abnormalities.

Follow-up of patients with brachioradial pruritus

The nine patients with BRP attained variable improvement of their symptoms with medications (gabapentin, naproxen, oral and topical steroids, combination topical capsaicin/lidocaine), with gabapentin providing the most relief. One patient underwent cervical epidural injections and acupuncture for BRP without benefit.

## Discussion

While it is unclear whether cervical radiculopathy is the major causative factor in BRP [1, 3, 18, 19], increased rates of cervical spine disease from C5-C8 have been described in BRP [3, 4, 9]. BRP has been reported in patients with cervical disc herniation, osteophytes, neural foraminal stenosis, spinal neoplasms such as ependymoma, post-traumatic syringomyelia, and following an incomplete traumatic cervical spinal cord injury (C6 motor and sensory level bilaterally) with C6-7 posterior instrumentation and fusion [1, 14, 19-22]. In Marziniak et al.’s study of 41 patients with BRP, 33 (80.5%) patients had stenosis of the intervertebral foramen or protrusions of the cervical disc that led to nerve compression [19]. The location of the nerve compression lesions correlated significantly with the dermatomal localization of pruritus (p<0.01). Similarly, all 11 of 22 patients with BRP who underwent cervical spine radiographs in Goodkin et al.’s study had evidence of cervical spine disease that could be correlated with the location of pruritus [18].

The cervical origin of BRP has also been supported by myriad treatments aimed at the cervical region with ensuing relief of pruritic symptoms, including cervical spine manipulation, physiotherapy, and cervical nerve root blocks [8, 9, 14, 19]. In Weinberg et al.’s case series of three patients with BRP (all female with an average age of 66 years and bilateral symptoms), CT-guided cervical epidural steroid injections were administered [8]. Two patients attained complete symptomatic improvement after one injection, while the third patient underwent three injections with mild-moderate relief. De Ridder et al.’s patient with BRP underwent two cervical epidural steroid injections that resolved the pruritis and improved C-fiber function from C6-8 on quantitative sensory testing [14]. Surgery for a cervical disc herniation, degenerative disease, or a spinal tumor has also been shown to assuage BRP symptoms [6, 8, 19, 20]. Binder et al. reported the case of a patient with pruritus, burning, and paresthesias involving the sixth cervical nerve root distribution of the dorsal forearm [20]. A cervical MRI demonstrated a disc herniation and intervertebral osteochondrosis at C5-6, and the patient underwent an anterior cervical discectomy/fusion (ACDF) at C5-6 with C6 nerve root decompression. The symptoms resolved within one week postoperatively. Another patient with BRP and multilevel cervical spondylosis on cervical MRI underwent an ACDF from C3-C7 with an immediate improvement of his symptoms [6]. The incidence of incidental degenerative spinal pathology in patients who undergo an MRI after the 4th decade ranges between 20-40%.

It has been proposed that BRP is caused by either peripheral nerve damage (induced by solar radiation) or from sensory pathways involving the spinal cord and/or cervical nerve roots (caused by cervical spine disease) [13]. A-delta and C-fibers both transmit the sensation of pruritus, with the nociceptor C-fibers transmitting impulses to the dorsal horn of the spinal cord and from there to the thalamus via the spinothalamic tract. In BRP, stimulation of the A-delta impulses may act in a negative feedback mechanism to shut off C-fiber stimulation which paradoxically potentiates the pruritic sensation [3]. BRP may result from the irritation of pruritus-sensitive neurons that leads to spontaneous firing of the damaged neurons, the loss of feedback mechanism for their descending inhibitory neurons, or loss of inhibitory interneurons leading to spinal hyperexcitability [19, 23]. Intense solar radiation may be responsible for the spontaneous firing of solar-damaged nociceptors causing the exacerbation of pruritis [4, 24]. Furthermore, as pain and itch sensations are transmitted by the same unmyelinated C-fibers, it has been suggested that there is a complex interaction between the nociceptive and pruritoceptive neurons in the spinal cord caused by compressive lesions which leads to the predominance of either the pruritic or pain sensation [1]. Without a prior history of soft tissue or nerve injury in our cases, we did not consider complex regional pain syndrome (CRPS) and causalgia. Patients with a past history of trauma were not included in our study.

The mechanism by which BoNT acts on neuropathic pain involves inhibiting the secretion of pain mediators (substance P, glutamate, and calcitonin gene-related protein) from nerve endings and dorsal root ganglia (DRG), decreasing local inflammation around the nerve endings, deactivating the sodium channel, and demonstrating axonal transport [25]. BoNT reduces TRPV1 expression by inhibiting plasma membrane trafficking. Capsaicin induces an initial hypersensitization followed by nerve desensitization through two mechanisms: (1) secretory efferent function of TRPV1 neurons release neuropeptides by exocytosis upon their depolarization; and (2) antidromic reflex stimulation of DRG neurons [26].

EDX has not been routinely done in the investigation of patients with BRP [14]. As nerve conduction studies measure conduction of myelinated nerves and itch fiber afferents are unmyelinated C-fibers, it has been opined that these studies are ineffective in diagnosing BRP [13]. However, needle EMG is valuable in detecting a cervical radiculopathy that is often associated with BRP. Cohen et al. performed EDX on seven patients (five men and two women with an average age of 58.3 years) with BRP [12]. Four (57%) patients had bilateral delay of the F responses of the median and ulnar nerves with latencies above 30 seconds that were considered by the authors to be diagnostic for cervical radiculopathy. In our series eight of the nine patients showed EDX evidence for cervical radiculopathy. In the one patient with negative EDX study, the MRI showed abnormalities in the cervical spine. It is possible that the dorsal root components were more severely involved, sparing the motor axons thus leading to absence of denervation/reinnervation changes.

## Conclusions

Our case series illustrates the importance of adding electrodiagnostic data to the clinical and MR imaging findings to arrive at the cause of BRP. The presence of MR and EDX findings supportive of cervical radiculopathy in all of our patients further substantiates the role of cervical spine disease in BRP. While the underlying pathophysiology is unclear, the discovery of selective neural pathways for itch and pain suggests the possibility that spinal root/cord lesions may lead to the predominance of pain or itch sensation. While BRP is a clinical diagnosis and may respond to symptomatic treatment of pruritus, the presence of additional neurological findings suggestive of a cervical root or cord lesion should prompt further workup including imaging studies of the cervical spine and, if indicated, EDX studies.
